# Apatinib alone or combined with radiotherapy in metastatic prostate cancer: Results from a pilot, multicenter study

**DOI:** 10.18632/oncotarget.22719

**Published:** 2017-11-28

**Authors:** Feng Zhao, Wei Tian, Ming Zeng, Jianling Xia, Honglin Hu, Xinbao Hao, Liangfu Han, Hao Liu, Yangke He, Xueqiang Zhu, Liang Liang, Rui Ao, Min Wei, Lili Deng, Yuquan Wei

**Affiliations:** ^1^ State Key Laboratory of Biotherapy and Cancer Center, West China Hospital, Sichuan University, and Collaborative Innovation Center for Biotherapy, Chengdu, Sichuan, PR China; ^2^ Cancer Center Hospital of University of Electronic Science and Technology of China and Sichuan Provincial People’s Hospital, Chengdu, Sichuan, PR China; ^3^ Operations Management Department, Hospital of University of Electronic Science and Technology of China and Sichuan Provincial People’s Hospital, Chengdu, Sichuan, PR China; ^4^ School of Medicine, University of Electronic Science and Technology of China, Chengdu, Sichuan, PR China; ^5^ Sino-America Cancer Center, Hainan Medial University, Haikou, Hainan, PR China; ^6^ Department of Radiation Oncology, ChangAn Hospital, Xi’an, Shanxi, PR China; ^7^ Ziyang People’s Hospital, Sichuan, Ziyang, Sichuan, PR China; ^8^ Sichuan Friendship Hospital, Chengdu, Sichuan, PR China

**Keywords:** apatinib, radiotherapy, metastatic prostate cancer, adverse events, PSA

## Abstract

**Background:**

To study safety and efficacy of apatinib in combination of radiotherapy in patients with symptomatic bony disease prostate cancer(SBPC), based on the potential synergistic antitumor activity between apatinib and Radiation Therapy (RT).

**Patients and methods:**

In phase I dose escalation part, 18 patients received apatinib dose at 250 mg every other day, 250 mg daily and 500 mg daily. In phase II part, the 250 mg daily cohorts were expanded to 20 patients in combination of RT (6 Gy/fraction, 5 fraction in total), one patient lost followed up and excluded the study, comparing with RT alone cohort with 10 patients, ratio of RT to RT + apatinib was 1 to 2. Evaluations included adverse events (AEs), prostate specific antigen (PSA) changes, radiographic evaluation and pain relief.

**Results:**

In phase I study, common apatinib-related AEs (arAEs) were fatigue, anorexia, hand foot syndrome, proteinuria, and hypertension (HTN). Grade 3arAEs included HTN, proteinuria, liver dysfunction. In phase II study, combination apatinib with RT cohorts, AEs events increased comparing with either apatinib alone or RT alone; at the same time, combination cohorts showed PSA declines of ≥50% in 12 patients, and stable disease in 6 patients. Combination cohorts had pain control significantly improved in both level and duration comparing with RT alone.

**Conclusions:**

In SBPC patients, apatinib at less than 500 mg daily dose as mono-therapy had tolerable toxicity. Apatinib at dose of 250 mg daily in combining with RT synergized pain control, the overall AEs were manageable. Further studies are needed in large sample size future trials.

## INTRODUCTION

Apatinib is an orally bioavailable anti vascular epidermal growth factor (VEGF) small molecule tyrosine kinase inhibitor. Apatinib was demonstrated activity to targeting vascular endothelial growth factor receptor-2 (VEGFR-2), leading down regulation the proliferation of vessel endothelium and inhibiting tumor angiogenesis. Studies have revealed that anti-angiogenesis drugs inhibit the growth of solid tumors [1]; this anti-angiogenic agent also shows wide potential efficacy in a variety of solid tumors including metastatic lung, colon, and breast cancer [2, 3].

Apatinib as single agent demonstrated improvement in OS in stage IV gastric cancer, in patients failed to the 2nd line chemotherapy, showed improve OS from 4.7 month to 6.5 month with HR 0.70, *p* = 0.156 comparing with placebo. Adverse events (AEs) associated with apatinib were often blood vesicles associated, such as HTN, fatigue, proteinuria, which were generally managed conservatively [4, 5].

Palliative Hypofractionated Radiation Therapy (HFRT), focusing on symptomatic disease site alone, was widely used in metastatic prostate cancer for pain control [6]. With improvement of imaging guided RT (IGRT) and treatment planning system, modern RT techniques were able to deliver larger dose per fraction with sharp dose falloff to surrounding organs at risk (OAR). The mechanism of the RT damaged the tumor cell were proposed at multiple levels, beside directly damage cells by breaking DNA double strains, HFRT also indirectly impacts on cancer by “normalizing” angiogenesis [7, 8]. Therefore, we hypothesized that given apatinib during RT may enhance the antitumor activity of apatinib in patients with metastatic prostate cancer. For those who have extensive pain disease, this and other treatments, including docetaxel, cabazitaxel, abiraterone, enzalutamide, and radium-223 chloride, showed symptomatic resolved without demonstrable effects on the serum prostate specific antigen (PSA) level. However, the potentially high toxicity and cost limited all patients to receive an simple and effective management to control pain. So there is a need for novel therapeutic approaches to provide durable symptomatic disease control. Accordingly, we performed a pilot study in patients with symptomatic bony metastatic adenocarcinoma of prostate to systematically assess apatinib at various doses given alone or in combination with external-beam radiotherapy (RT). We aim to determine the dose limited toxicity (DLT) and recommended dose of apatinib when combine with RT as palliative approach [9–14].

## RESULTS

### Patients

Fifty patients were enrolled at 3 sites: 47 were eligible and received treatment between May 2016 and September 2016. Patient demographics and baseline disease characteristics are listed in Table 1. Patient average ages at different group are between 68 to 70 years old. The majority performance status are ECOG 0 to 1, only few with ECOG level 2. Bony metastases were common diseases, with less has nodes disease or lung metastases. Among those with bony metastases, the metastases sites ranges from 1 to 5, those more than 5 bony metastases were excluded in the study. The primary diseases were treated with either surgery, accounted for 17, 36%, or primary radiation, 3, 6.3%. 58% patient with stage IV disease without primary site treatment.

**Table 1:** Patient Characteristics

Characteristic	0	125	250		500
	RT (*n* = 6)	–RT (*n* = 10)	–RT (*n* = 19)	RT (*n* = 6)	–RT (*n* = 6)
Age (years)					
Median	68	70	69	68	68
Range	59–83	56–81	59–77	56–82	61–78
ECFOG PS					
0	5	6	14	6	5
1	1	3	4	0	1
2	0	0	1	0	0
n/a		1			
Selected tumor lession					
LN	0	2	5	1	2
Lung	1	0	4	0	2
primary treated	3	2	8	2	4
bone Lesions (no)					
Median	3	2.5	5	3	4
Range					
Serun PSA					
Median	89	108	53	69	53
Range	<1–109	<1–344	21–149	<1–167	19–131
Selected prior Therapy					
Suery	3	2	7	1	4
RT			1	1	
ADT	4	1	1	8	1
Chemotherapy	1			2	

RT, Radiotherapy; ECOG, Eastern Cooperative Oncology Group; PS, performance Status, chemotherapy, Dpcetavel; ADT, androgen androgen deprivation therapy.

### Apatinib alone showed PSA suppression efficacy

**Figure 1:**
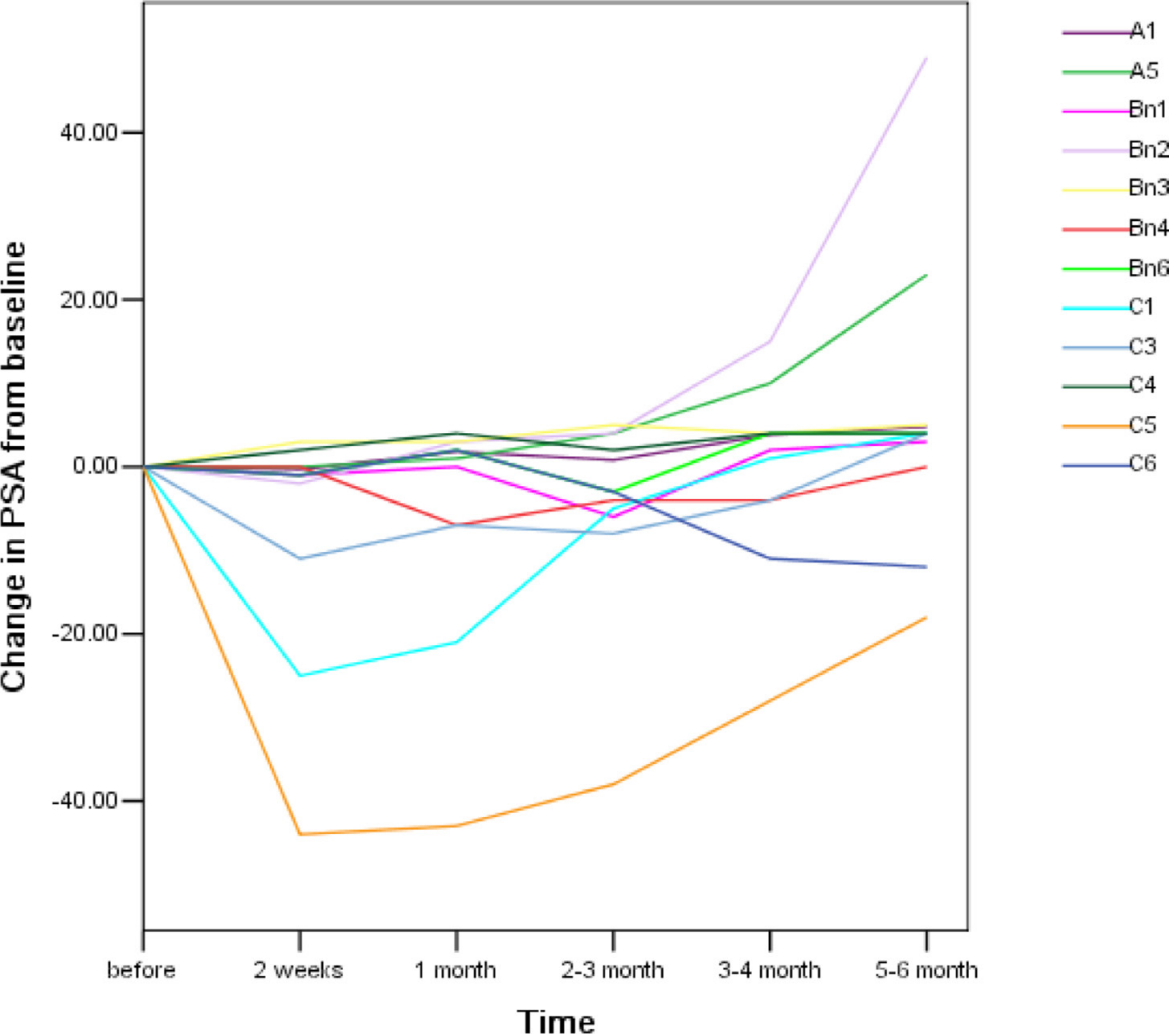
Spaghetti plots of change in PSA from baseline The change in PSA with time plots started with pretreatment level. Among 3 different doses of apatinib were ploted in one figure. A, B and C represented with 250 mg every other day group, 250 mg daily group, 500 mg daily accordingly. The number in each group represented individual patient. Total PSA measure at different time peroid after apatinib treatment. Those received ADT, total 6 patients, excluded in the plots.

In phase I study, the anti-tumor activity by apatinib were measurable, some patient with PSA decline (Figure 1), 500 mg cohort showed PSA decline level >50%. Trend of dose response of apatinib with more PSA decline demonstrated (Figure 1). The duration of PSA decline is about 2 to 3 month, then rebound of PSA found even with continuation of apatinib. Higher dose of apatinib did not showed longer duration of decline PSA. In the 250 mg every other day group, the median number of apatinib doses per patient was 14 (range: 12–15). Patients in 250 mg cohorts received a median of 30 (range: 29–31). Patient in 500 mg cohort received a median of 58 (range: 56–62). The PSA changes with time illustrated in the spaghetti plots, Figure 1. The figure plots apatinib alone cohorts and PSA changes, there were 3 patients with obvious and transit declining PSA, with time, PSA gradually increased, even with continuation of apatinib. 2). In phase II study, the greater PSA suppression found in combined RT with apatinib cohorts (Table 2). 12 (63%) of combined cohorts showed the PSA decline >50%; on the other hand, 5 (50%) patient of RT alone cohorts showed PSA decline >50%. Apatinib alone cohorts showed no patient with PSA decline >50% (Table 2). Those with nodes and lung disease, the follow up study did not showed initial response or showed stable disease by PCWG1 criteria or RECIST.

**Table 2:** PSA Decline^*^

Characteristics	250 mg			0
	–RT *n* = 6 (%)	RT *n* = 19 (%)	±RT *n* = 25 (%)	+RT *n* = 10 (%)
PSA decline				
anytime	1 (16)	15, (78.9)	16 (64)	6 (60)
>50%	0	12 (63)	12 (48)	5 (50)
PSA decline				
duration	2 month	2–6 months	n/a	2–6 month
ADT	1	4 (21)	5 (20)	4 (40)

*ADT patients were excluded from PSA decline analyses.

### Apatinib showed acceptable toxicity with or without combination with RT in SBCAP

Twenty nine patients were initially treated with RT with or without apatinib. The adding of apatinib did not worsen the toxicity profile (Table 3A and 3B). 18 patients were initially treated with escalating doses of apatinib from 250 mg every other day (125 mg average), to daily (250 mg average) and to twice a day (500 mg average). There were no DLTs during the 4-week assessment period. Since the MTD was not reached per previous report [2, 4, 5], best tolerance dose of 250 mg cohorts were expanded to 30 patients in phase II study. Treatment-related AEs were common, with the majority being grade 1/2 (Table 3A and 3B). Most of the treatment-related AEs were arAEs in each cohort, and the majority of arAEs were grade 1/2 (Table 3). Common (≥15%) arAEs of any grade in the RT ±250 mg group were hand-food syndrome, HTN, Liver function changes, neutropenia, fatigue. Other common treatment-treated AEs were mucositis, thrombocytopenia, hematuria, anorexia, dizziness, fever, vomiting and skin ulceration. 13 patients (35.1%) reported arAEs of grade 3, most common were hand-food syndrome, LFT abnormal, HTN, mucositis and skin ulceration (5.4%, all grade 3). There were no reports of bowel perforation, pneumonitis, cardiomyopathy or neurologic arAEs. RT did not add grade 3 toxicity in 250 mg patient cohorts. 3 patients discontinue treatment due to arAE, others recovered once the drug was on hold and symptomatic or conservative manage the patients. After one to two weeks treatment break, start restart with one level step down dose and continue for the rest of the course. None of treated patient with persistent grade 3 arAE at time of 6 month. Three patients (6.4%) discontinued the study due to arAEs (Table 1). Of three discontinuations due to AEs, one was caused by grade 3 hand-food syndrome, one by grade 3 hepatitis, one grade 3/4 by neutropenia, the discontinuation were determined by patients, none of them require hospitalization for the condition. No treatment related death found in this cohort.

**Table 3A:** Safety

	0				125			
	RT (*n* = 10)				–RT (*n* = 6)			
Adverse event	Gd 1* (*n*, %)	Gd 2 (*n*, %)	Gd 3 (*n*, %)	Total	Gd 1* (*n*, %)	Gd 2 (*n*, %)	Gd 3 (*n*, %)	Total
Fatigue	–	–	–	–	3	–	–	(3, 50%)
Anorexia	–	–	–	–	1	1	–	(2, 33.3%)
Hand-foot syndrome	–	–	–	–	2	–	–	(2, 33.3%)
Proteinuria	–	–	–	–	1	1	0	(2, 33.3%)
Hypertension	–	–	–	–	–	1	1	(2, 33.3%)
Neutropenia	1	–	–	(1, 10%)	1	1	0	(2, 33.3%)
Bilirubin increased	–	–	–	–	2	1	–	(3, 50%)
Transaminase increased	–	–	–	–	1	–	–	(1, 16.6%)
Mucositis	1	1	–	(2, 20%)	–	2	–	(2, 33.3%)
Thrombocytopenia	–	–	–	–	1	–	–	(1, 16.6%)
Hematuria	–	–	–	–	1	–	–	(1, 16.6%)
Dizziness	–	–	–	–	1	–	–	(1, 16.6%)
Fever	–	–	–	–	–	–	–	–
Diarrhea	1	–	–	(1, 10%)	1	–	–	–
Skin ulceration	–	–	–	–	–	–	–	–
Vomiting	1	–	–	(1, 10%)		–	–	–
Dyspnea	–	–	–	–	–	1	–	(1, 16.6%)
Total events	4	1	0	5	15	8	1	24

GD 1*, grade1 toxicity

**Table 3B:** Safety

–RT (*n* = 6)				250 mg				500			
				RT (*n* = 19)				–RT (*n* = 6)			
Gd 1* (*n*, %)	Gd 2 (*n*, %)	Gd 3 (*n*, %)	Total	Gd 1* (*n*, %)	Gd 2 (*n*, %)	Gd 3 (*n*, %)	Total	Gd 1* (*n*, %)	Gd 2 (*n*, %)	Gd 3 (*n*, %)	Total
2	–	1	(3, 50%)	1	1	1	(3, 15.7%)	3	2	–	(5, 83.3%)
2	1	–	(3, 50%)	2	–		(2, 10.5%)	2	3	–	(5, 83.3%)
1	1	1	(3, 50%)	2	2	1	(5, 26.3%)	2	1	1	(4, 66.6%)
2	1	1	(4, 66.6%)	1	3	0	(4, 21.0%)	1	1	0	(2, 33.3%)
–	2	0	(2,33.3%)	1	2	1	(4, 21.0%)	1	2	1	(4, 66.6%)
1	1	–	(2,33.3%)	2	3	–	(5, 26.3%)	2	0	1	(3, 50%)
2	2	–	(4, 66.6%)	3	2	2	(7, 36.8%)	2	1	–	(2, 33.3%)
2	1	–	(3, 50%)	2	3	–	(5, 26.3%)	1	1	1	(3, 50%)
3	1	–	(4, 66.6%)	3	3	2	(8, 42.1%)	1	1	–	(2, 33.3%)
1	1	–	(2,33.3%)	2	2	–	(4, 21.0%)	2	2	–	(4, 66.6%)
1	–	–	(1, 16.6%)	–	–	–	–	2	–	–	(2, 33.3%)
–	–	–	–	1	–	–	(1, 5.2%)	1	1	–	(2, 33.3%)
1	–	–	(1, 16.6%)		–	–	–	–	–	–	–
–	–	–	–	1	2	–	(3, 15.7%)	1	–	–	(1, 16.6%)
–	–	–	–	2	–	–	(2, 10.5%)	–	–	–	–
–	–	–	–	–	–	–	–	1	–	–	(1, 16.6%)
–	1	–	(1, 16.6%)	–	–	–	–	–	–	–	–
15	12	3	30	23	22	7	52	19	12	4	35

### RT combined with apatinib improve pain control duration

**Figure 2:**
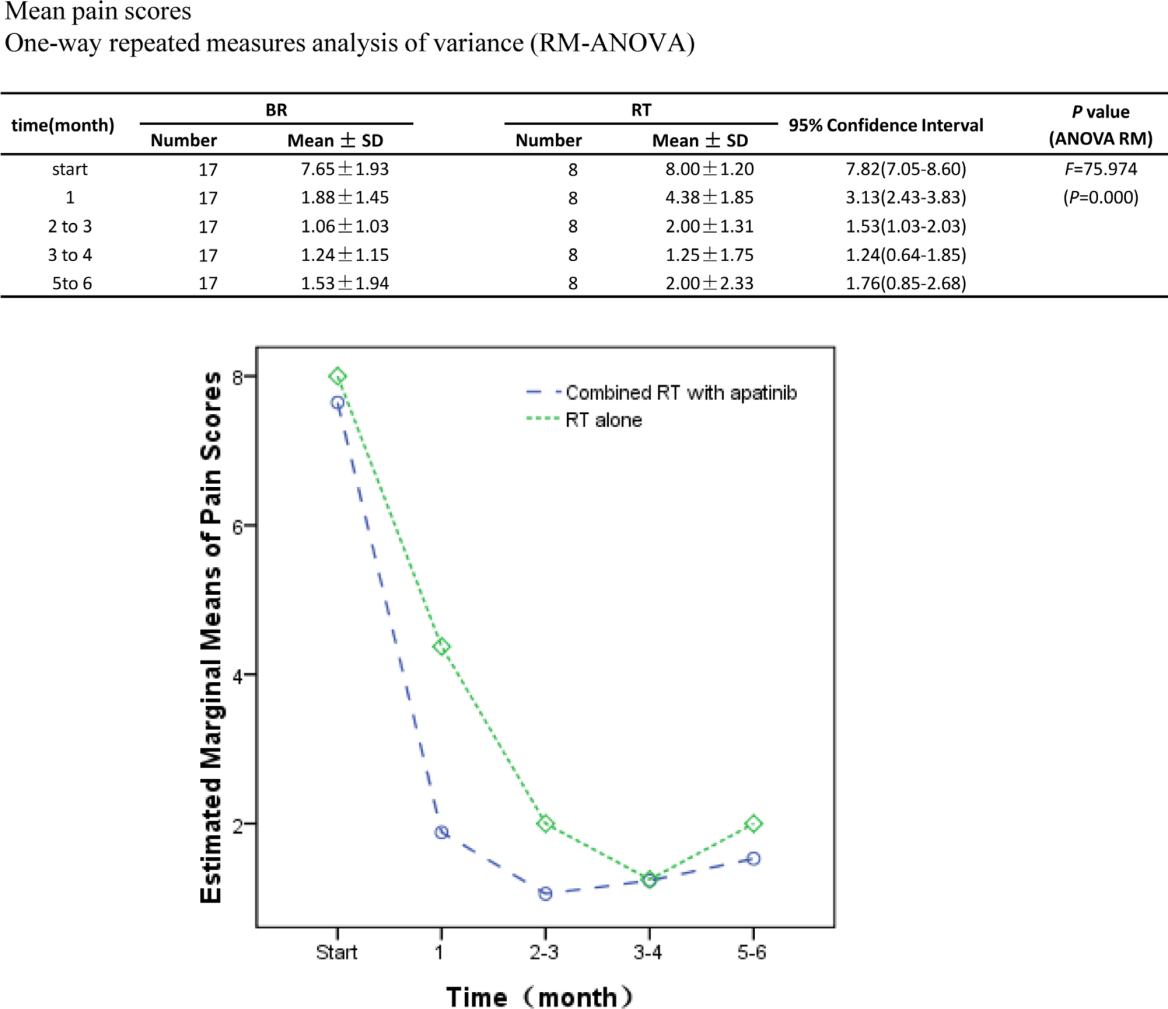
Pain evaluation changes with time The pain level was evaluated from RT with or without apatinib cohorts. Average pain levels with time were ploted. The averages pain level from two cohorts were analyzed. Significant difference fund in the pain level between combined apatinib with RT and RT (*F* = 4.588, *p* = 0.043). The estimated marginal mean pain with combined apatinib with RT (2.67, 95% CI 2.20–3.14) was significantly less compared to RT alone (3.53, 95% CI 2.85–4.21). In both groups, those took pain medicines during the study were excluded in the study, the eligible patients in combined RT with apatinib and RT alone were 17 and 8 accordingly.

During the phase I study, the pain evaluation showed no significant difference in pain control at various dose of apatinib. Thus, the further analyses were excluded apatinib alone cohorts. Pain measurement was only conducted in combination cohorts. RT itself provided great pain control. Adding apatinib to RT, the pain level was further decreased, and pain control duration was enlonged. Statistic analysis showed the significance between the two groups (Figure 2). Between the two groups，no significant radiographic response within the study follow up period. Two patients with progressive disease in lung, one with progressive disease in the lymph node.

## DISCUSSION

In current study, we made a systematic evaluation of the safety and efficacy of apatinib as mono-therapy or in combination with radiotherapy in symptomatic bony metastatic CAP patients. Patients in all cohorts, including 250 mg with or without radiotherapy, experienced frequent (all grades, 59%; grade 3/4, 31.9%), but not unexpected apatinib related arAEs, which consisting mainly of Fatigue, HTN, abnormal LFT and hand-food syndrome. Within the 4-week assessment period, no DLTs were noted, grade 3/4 AEs were also noticed beyond the DLT window, however, it was all short lasting, no long term sequel noticed once discontinuation of the apatinib. The arAEs were generally managed with supportive care, less frequently managed by corticosteroids. 3 patients need to change their anti hypertension medicine to maintain blood pressure normally. No long term need for supportive care related to arAE. Early recognition and adequate intervention of arAEs, particularly hypertension, proteinuria is necessary. Comparing with 500 mg, 250 mg was chosen to carry out the phase II study was due to better tolerance and manageable safety profile. This group was expanded to 20 metastatic prostate patients, one discontinued the study, while the rest of 19 patients were analyzed.

Clinical activity was assessed by both PCWG1 and RECIST guidelines and pain evaluation. Apatinib had limited activity in controlling the disease, particular visceral disease by PCWG1 and RECIST guideline. No PR or CR found in the cohorts. So does the pain control in apatinib mono-therapy at different dose level.

However, in the apatinib in combination with RT cohort, PSA decline noticed cross the board, also more PSA declining >50% noticed, irrespective of prior exposure to chemotherapy. These PSA data, taken together with the observation that, of 25 tumor-evaluable patients receiving 250 ± RT, the majority had stable disease, suggesting potential clinical antitumor activity with disease control in such level to subgroup patients. PSA declines further suggest a direct antitumor effect of apatinib. In addition, 250 mg apatinib showed significant pain relief in combination of RT, comparing with RT alone, the pain relief lasted much longer (*P* < 0.05).

Apatinib is a compound derived from valatinib, is an oral, and highly potent inhibitor of vascular endothelial growth factor receptor-2 (VEGFR-2) tyrosine kinase targeting the intercellular ATP-binding site of the receptor, down regulating the phosphorylation, and subsequent downstream signaling.

In the phase III study of apatinib, patients were randomized to receive oral apatinib (850 mg once daily) or placebo at a ratio of 2:1. Apatinib significantly improved median overall survival (OS) time (6.5 months vs. 4.7 months; *P* = 0.015) and PFS time (2.6 months vs. 1.8 months; *P* < 0.001) in metastatic gastric cancer patients who progressed on two or more lines of chemotherapy. In addition, apatinib showed potent activity against lung, breast and colon cancer and malignant fibrous histiocytoma [2–5, 15]. Apatinib showed manageable toxicity as reported before in our study, hand-foot syndrome, proteinuria, and hypertension were the most common treatment-related non-hematologic adverse events which is similar to other anti-angiogenic agents. Serious side effects, such as gastrointestinal massive hemorrhage and perforation were also reported [4, 5].

Our findings have a promising potential for clinical application, with manageable toxicity profile, small tyrosine kinase inhibitor (TKI) molecules are able to maintain pain control either at Radiation treated site or RT naive sites in SBCAP. To our knowledge, this is the first study to report that the small TKI molecules have a potential role in pain control. The mechanism remains to be explored. We proposed two possible pathways, 1. Blood Vessel “normalization” related pathway. Apatinib has multi-targets, besides antitumor activity in VEGFR-2 [7, 16], it also targets on blood vessel, possibility of prolonging pain duration through normalization of vessel proposed here. It is well known that newly formed tumor blood vessels are fragile and extremely sensitive to ionizing radiation. Various lines of evidence indicate that irradiation of tumors with high dose per fraction, not only kills tumor cells but also causes significant damage in tumor vasculatures. Such vascular damage and ensuing deterioration of the intratumor environment then cause ischemic or indirect/secondary tumor cell death within a few days after radiation exposure, indicating that vascular damage plays an important role in the response of tumors to RT [15, 17–20]. A finding of TKI molecules synergized with RT was demonstrated before in mice. El Kaffas reported that treatments where sunitinib was combined with radiation demonstrated a significant increase in murine vascular flow signal. This was accompanied with a significant increase in cell death when compared to radiation or sunitinib alone [21]. Clinical application of combined TKI with RT was also report in solid tumor in human [22]. Anti vascular endothelial growth factor (VEGF) monoclone antibody combined with chemotherapy were used in human malignancies as well [23, 24]. 2). Direct antitumor activity pathway, the fact that decline in PSA in small subgroup metastatic adenocarcinoma patients, suggesting its antitumor activity. This activity last for less than 4 weeks, PSA rebounded in all initial response patients after 4 weeks period. PSA rebound suggested establishment of antitumor activity resistance. Antitumor activity resistance measured solo by PSA happened quicker comparing with that in other malignancies, such as breast or gastric cancer [2–5]. In our study, the interaction of apatinib and RT for pain control was found to be synergistic, this synergistic pain control lasted for several months. What is the optimal combination conditions and how long the synergistic result would last remain to be further studied.

Our finding needs to be explained with caution. First, this was a pilot study: sample size was small, which constrained the significance of the finding, there was also potential selection biases. Second, the follow-up was short and cohort had in-homogeneity patients, such as without group patient per castration resistance et al. In a prospective setting, the patients could be stratified by different prognostic clinical variables in an effort to better elucidate the role of apatinib as mono-therapy or in combination with RT in symptomatic control in SBCAP.

## CONCLUSION

We conducted phase 1 and II study in SBPC patients using apatinib agent. In phase I, Apatinib dose escalation from 250 mg every other day, to 250 mg daily, to 500 mg daily. Overall, the apatinib at less than 500 mg daily dose as a mono-therapy had tolerable toxicity, common arAE were fatigue, anorexia, proteinuria, hypertension. Toxicity data were consisted with previous report in other malignancy patients. During phase I study, there was antitumor activity measuring with PSA decline in small portion patients, which subgroup PSA declining sensitive patients need further investigation. In phase II, Apatinib at dose of 250 mg daily in combining with RT synergized pain control, the pain control in level and duration might be in favorable to combination therapy with statistic significance. Overall AEs happened more often in combination group, but they were short lasting and manageable. Further studies are needed in large sample size future trials.

## PATIENTS AND METHODS

### Patients

All adenocarcinoma of the prostate (CAP) was confirmed histological, and the extent of disease was documented radiographically by bone scan and computed tomography, those were diagnosed with SBCAP (rising PSA or progression on scans with symptomatic lesion(s) in the bony) were included in the study. Other including factors were the following a life expectancy of >12 weeks, an Eastern Cooperative Oncology Group (ECOG) performance status of less than 2, and adequate hematologic, hepatic, and renal functions. Excluding factors were: patients with radiation-induced diarrhea within 12 months of study entry or with prior colitis or irritable bowel syndrome were excluded. Other excluding criteria were autoimmune disease (except for vitiligo) requiring systemic steroids or immunosuppressive agents, other prior malignancy within 5 years, active infection. All patients gave informed consent before enrollment. The study was conducted according to the principles of the Helsinki Declaration. The protocol was approved by the Institutional Review Boards.

### Study design and treatment

**Figure 3:**
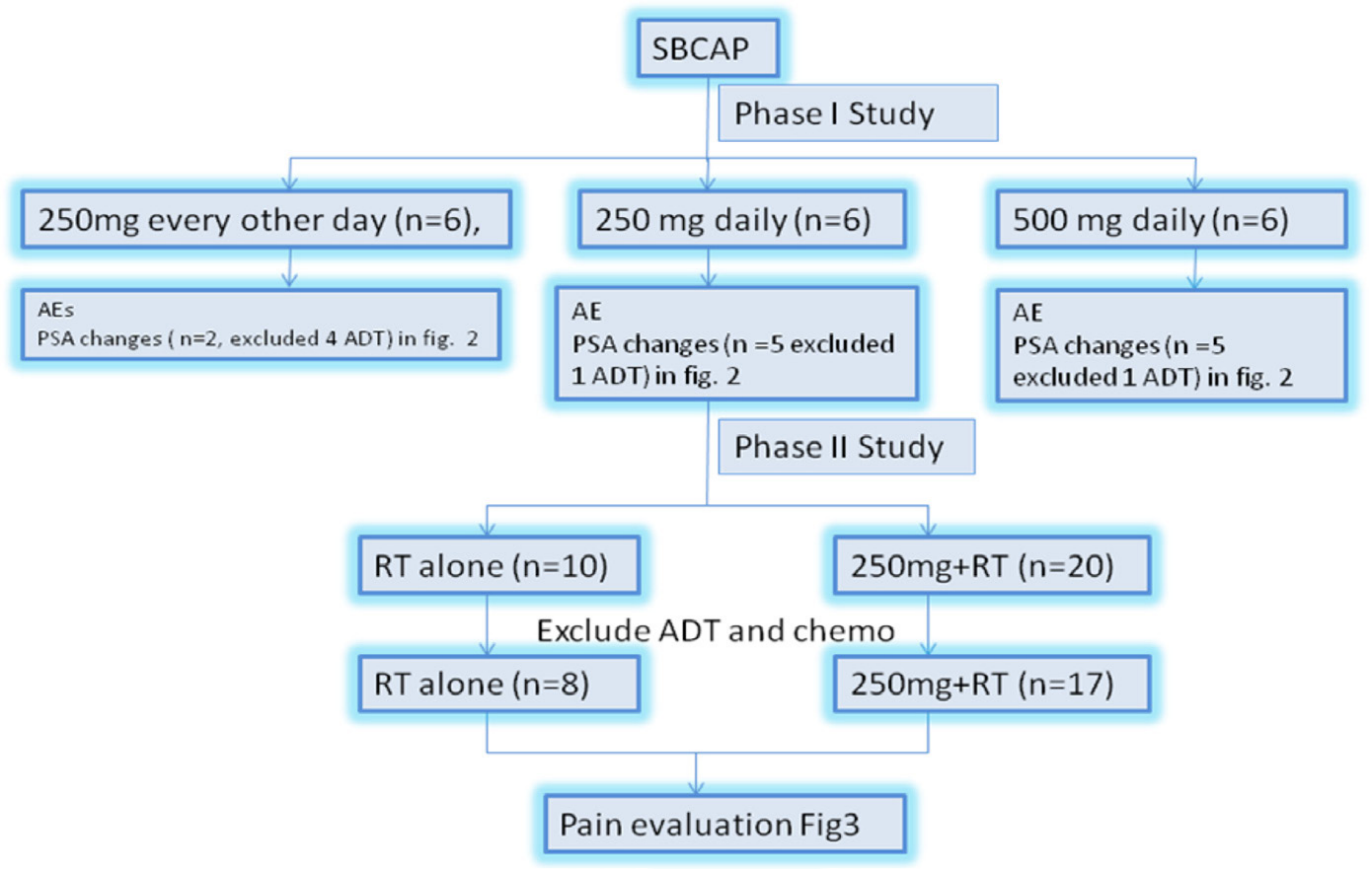
The study schema SBCAP, symptomatic bony disease prostate cancer; AE, adverse events; PSA, prostate-specific antigen.

This was a phase I/II, non-randomized, open-label, multicenter study ARCAP study (ClinicalTrials.gov identifier: NCT02998242). In the phase I part eligible patients (≥6 patients per cohort) received Apatinib (Hengrui Pharmaceutical Co. Ltd) at 250 mg every other day, 250 mg and 500 mg daily. Dose escalation in phase I occurred after all six patients in the preceding cohort received at least 4 week dose treatment and were observed for an additional 2 weeks with no more than one of the six patients experiencing a dose-limiting toxicity (DLT) during this 6-week period. The DLT was defined as a grade 3/4 Apainib-related AE (arAE) or other grade 3/4 treatment-related AE, which required surgical intervention or did not resolve to ≤ grade 2 within 14 days of the start of apatinib therapy. Dose escalation continued until the last monotherapy-dose cohort was enrolled or the maximum tolerated dose (MTD, defined as the highest dose at which no more than one of the six patients experienced a DLT) was identified. Once the mono-therapy cohorts were fully enrolled, patients were assigned to RT(radiotherapy) ±250 mg daily, phase II study (Figure 3). Radiotherapy was given focally at five fractions of total dose 30 Gy per target symptomatic bone lesion for up to five bone lesions per patient over one or two weeks duration. RT was given on the day for the first apatinib dose. IMRT technique recommended with dose constrain per AAPM TG 101 [25]. Target lesions had to be ≥10 mm long in at least one direction when measured by radiologic imaging or Bone scan positive for update. The timing of RT delivery was designed to provide peak level of apatinib before RT completion to maximize the synergistic effect. Apatinib 250 mg daily, on the 1st day of RT. The apatinib will continue till pain developed.

No retreatment to previously treated area with RT was allowed. After the initial treatment period (days 1 to 6 months), patients were first followed up at two weeks, then every 4–6 weeks for 3 months and subsequently every 3 months for 9 months or until disease progression, intolerance, or death. Patients who withdrew from the study due to disease progression or who completed all planned study visits were followed for survival every 3 months.

### Assessments

AEs including apatinib-related AEs (arAEs) were based on assessments by investigators of patients treated between the first dose, in two weeks and 30 days after the last fraction of radiation treatment. An arAE was defined as a treatment-related AEs. AEs using the NCI Common Terminology Criteria for Adverse Events, version 3.0 [26].

The protocol defined guidelines for evaluation and treatment of arAEs of the cardiac vascular, GI, skin endocrine glands. arAE management consisted of discontinuous treatment or symptomatic methods. Corticosteroids given orally or intravenously were also used at the investigators’ discretion. No dose reductions were allowed. For a grade 2 drug-related skin arAE or grade 3 skin arAE (regardless of causality), apatinib administration was delayed until its resolution to ≤ grade 1. Apatinib administration was permanently discontinued for any related AE of ≥ grade 3 or any other AE of ≥ grade 4.

Pains were evaluated according to the Pain Stages of Change Questionnaire (PSOCQ) [27–30]. Those patients that have chronic pain before diagnosed with SBCAP were excluded in the study. Evaluation was done through the Universal pain assessment tool.

Antitumor effects were assessed by serum PSA status using criteria consistent with guidelines of the Prostate Cancer Clinical Trials Working Group 1 (PCWG1) [1], the standard when the study was being designed, and by tumor status using Response Evaluation Criteria in Solid Tumors (RECIST) for soft tissue disease [26]. PSA assessments were performed on days 1, two weeks, 1 month, and 3 months thereafter. Tumor assessments were carried out on day 30 and every 3 months thereafter. Both the decline in PSA to ≥50% from baseline (PSA decline) and tumor response, as determined by investigators, were confirmed by repeat assessments at 2 weeks after initial dose and 4 weeks or later after the initial assessments. The PSA decline was calculated by comparing the decline in post-therapy PSA concentration to baseline. End points included PSA decline by 2 weeks, one month, every 2–3 month. Assessment included PSA decline at any time, tumor response at any time, time to and duration of tumor response.

### Statistical considerations

In phase I portion of the study, the objective of this study was to determine the safety of apatinib alone or in combination with RT. In the phase II portion of the study, the objective was to determine clinical antitumor activity based on PSA, pain response and radiologic response to the apatinib alone or combination with RT .

In the phase I study, the initial sample size of 18 patients was based on the design of the dose escalation for safety. In the phase II study, there were to be 19 assessable patients treated with the combination of apatinib with stereotactic body radiation therapy (SBRT) and 10 treated RT alone, of 29 patients, chemotherapy-naïve 27; there were 5 receiving ADT, 24 hormone treatment naïve. Patients treated with chemo and ADT were excluded in subsequent pain evaluation analyses. Statistical analysis was performed using SPSS 17.0 Statistical Software. The repeated measures analysis of variance (RM-ANOVA) was used to assess statistical significance. The estimated marginal means are also reported with 95% confidence interval (CI). The two-tailed probability value *P* < 0.05 was considered statistically significant.
